# Supplementary material for: Oxidized Carbon-Based Spacers for Pressure-Resistant Graphene Oxide Membranes
Source: Membranes (Basel). 2022 Sep 26;12(10):934. doi: 10.3390/membranes12100934 (PMC9612342; doi:10.3390/membranes12100934)
Supplement: Supplementary file 1 [file membranes-12-00934-s001.zip › membranes-1916109-supplementary.pdf]

Supporting Information

Oxidized Carbon-based Spacers for Pressure Resistant Graphene Oxide Membranes

Ekaterina A. Chernova[a], Konstantin E. Gurianov[a], Dmitrii I. Petukhov[a,b], Andrei P. Chumakov[c], Rishat G. Valeev[d], Victor A. Brotsman[b], Alexey V. Garshev[b], Andrei A. Eliseev[a,b]

a. Department of Materials Science, Lomonosov Moscow State University, 1-73 Leninskiye gory, Moscow, 119991, Russia

b. Department of Chemistry, Lomonosov Moscow State University, 1-3 Leninskiye gory, Moscow, 119991, Russia

c. ESRF – The European Synchrotron. 71, Avenue des Martyrs, 38043 Grenoble, France

d Udmurt Federal Research Center of the Ural Brunch of Russian Academy of Sciences (UdmFRC of UB RAS), Izhevsk, st. them. Tatiana Baramzina 34, 426067, Russia

Corresponding Authors

Ekaterina Chernova: E-mail: chernova.msu@gmail.com

Andrei Eliseev: E-mail: eliseev@inorg.chem.msu.ru

S1. Characterization of fullerenols

The Fourier-transform infrared (FT-IR) spectra were registered within the wavenumber range of 400–4000 cm^{-1} (KBr pellet, resolution of 2 cm^{-1}) using an FT-IR IRAffinity-1 spectrometer (Shimadzu, Japan). UV/Vis absorption spectra were recorded in distilled water solutions with the use of AvaSpec-2048 fiber optic spectrometer (spectral range 200–1100 nm, optical resolution 2.4 nm, Avantes, Netherlands) equipped with the AvaLight-DH-S-Bal light source (200–2500 nm range, deuterium and halogen lamps, Avantes, Netherlands) at room temperature. TG/DSC thermogramms were recorded using STA 409 PC Luxx simultaneous thermal analyser (NETZSCH, Germany) combined with quadrupole mass spectrometer QMS 403C Aëolos (NETZSCH, Germany) over the temperature range 40–800 ºC in argon atmosphere at a heating rate of 10 °C min^{-1}. Dynamic light scattering (DLS) analysis was performed using Zetasizer Nano ZS instrument.

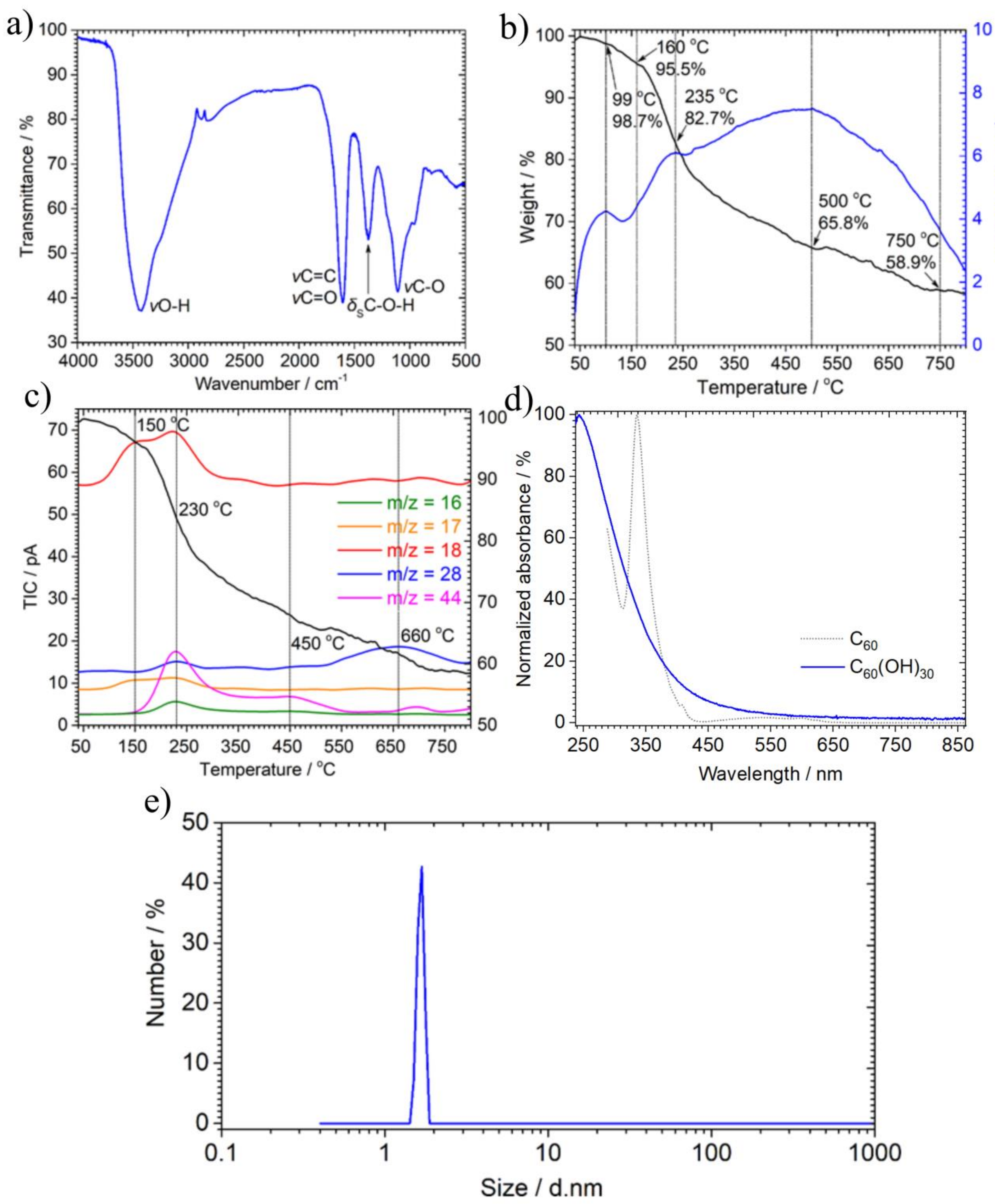

Figure S1. a) FT-IR spectrum of the fullerenols (KBr pellet, resolution of 2 cm^{-1}): IR (KBr pellet, cm^{-1}): 3424 (br, OH), 1607 (br, C=C), 1374 (br, C–O–H), 1109 (br, C–O). b) TGA and DSC curves for fullerenol. c) TGA–MS data for fullerenols; d) UV-vis absorption spectra for water solutions of fullerenols, and parent toluene solution of fullerene C_{60}. f) DLS analysis of fullerenols in water (0.1 wt %).

Table S1. Estimated number of bound water and –OH groups in fullerenol 1 based on TGA results.

Compound	Weight loss (temperature range, °C), %				Estimated number	
					bound water	–OH groups
	l_1 – loss due to volatiles	l_2 – loss due to bound water	l_3 – loss due to detachment of the OH groups	l_4	$\frac{M(C_{60}) * \boldsymbol{l_2}}{M(H_2O) * \boldsymbol{l_4}}$	$\frac{M(C_{60}) * \boldsymbol{l_3}}{M(OH) * \boldsymbol{l_4}}$
$C_{60}(OH)_n{\cdot}H_2O_m$ (**1**)	1.1 – 1.4 (40–95)	2.7 – 3.7 (95–150)	37.3 – 40.8 (160–750)	54.4 – 58.5 (>750)	1–3	26–32

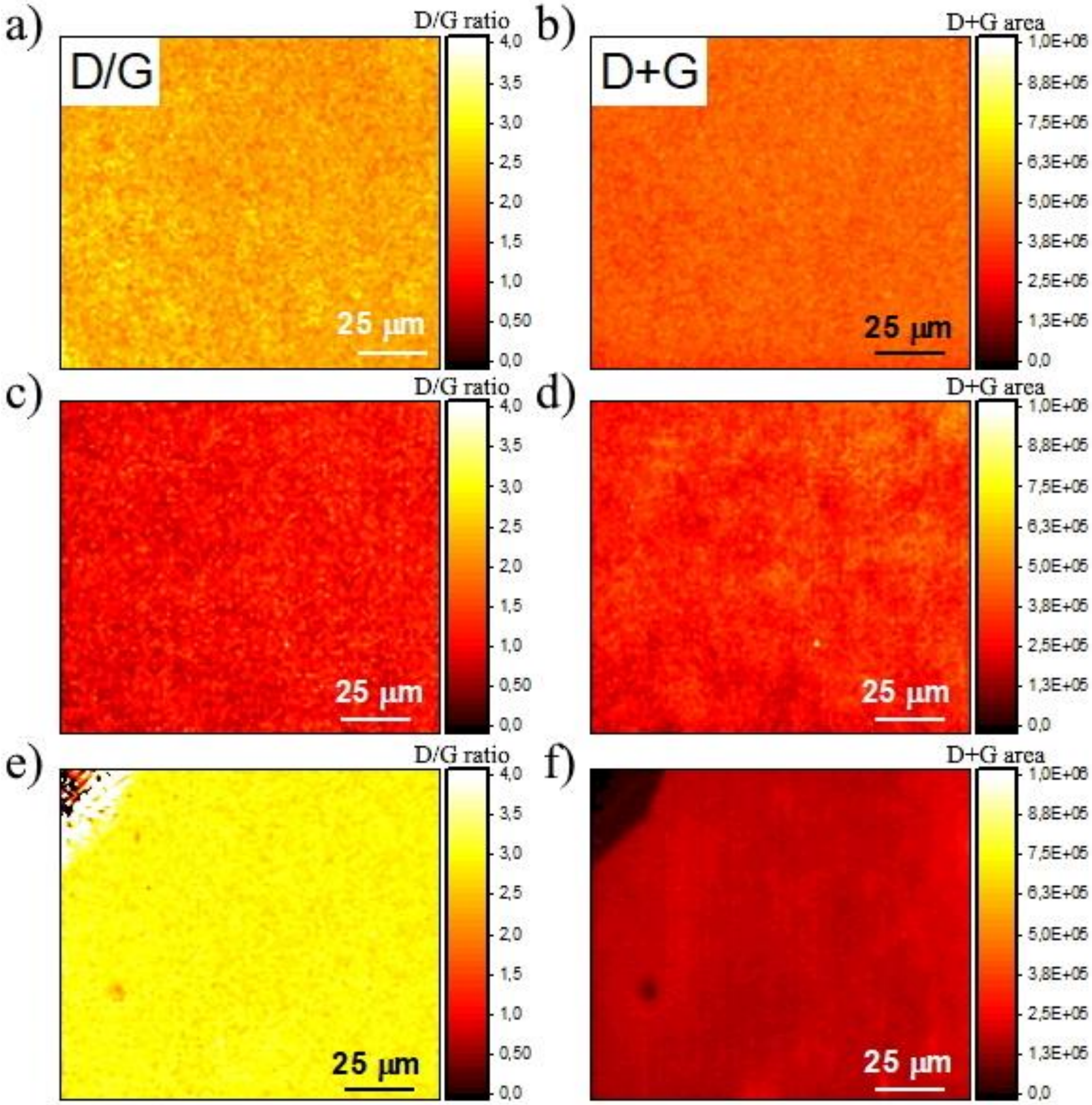

Figure S2. Raman maps of intensity distribution of the D/G ratio and D+G signals for: a, b) the MFGO reference membrane and intercalated composite membranes: c, d) $C_{60}(OH)_{30}$@MFGO-20% and e, f) CNTGO@MFGO-20%

S3. XPS results

Fitting of C1s spectra was performed according to the presence of C-C bonds network (~284.6 eV, carbon atoms fully coordinated to C or H), C-O (~286.6 eV, single oxygen-coordinated carbon in hydroxyl- or epoxy-groups) and C=O (~288.4 eV, double oxygen-coordinated carbon in aldehyde or ketone groups) [1]. We avoided quantitative determination of COOH groups (~289.4 eV) because of their relatively low concentration and overlapping with C=O component in the spectra. Also, due to insufficient spectral resolution, the deconvolution of C-C band into sp^3-C and sp^2-C was omitted. The O1s region was fitted accounting O=C (~531.0 eV) and H-O-C (~532.5 eV) components.

Table S3. XPS peak assignment for composite membranes based on graphene oxide

Sample	Bond	Peak position, eV	FWHM	Bond contribution, %	C-C/(C-O + C=O)	C/O
CNTGO	C-C	284.6	1.695	52.38	1.10	1.98
	C-O	286.81	1.695	40.45		
	C=O	288.80	1.695	7.17		
	O-C	532.75	2.010	82.86		
	O=C	531.20	2.010	17.14		
CNTGO@MFGO-20%	C-C	284.6	1.579	38.79	0.63	1.83
	C-O	286.81	1.579	55.37		
	C=O	288.67	1.579	5.84		
	O-C	532.58	1.909	87.67		
	O=C	531.15	1.909	12.33		
MFGO (reference)	C-C	284.6	1.628	34.46	0.53	1.81
	C-O	286.76	1.628	60.60		
	C=O	288.76	1.628	4.94		
	O-C	532.55	1.848	87.35		
	O=C	530.98	1.848	12.65		
$C_{60}(OH)_{36}$	C-C	284.6	1.594	50.21	1.01	1.52
	C-O	286.82	1.594	38.23		
	C=O	288.68	1.594	11.56		
	O-C	532.30	1.944	55.82		
	O=C	531.04	1.944	44.18		
	S2p	No				
MFGO@$C_{60}(OH)_{36}$-20%	C-C	284.6	1.641	37.48	0.60	1.88
	C-O	286.91	1.641	56.93		
	C=O	288.84	1.641	5.59		
	O-C	532.66	1.889	85.17		
	O=C	531.15	1.889	14.83		

S4 GIWAX data

Table S4. Interlayer spacing and FWHM for composite membranes

Sample	RH=0%		RH=100%	
	d-spacing, nm	FWHM	d-spacing, nm	FWHM
MFGO	0.7732	0.0765	1.1531	0.0659
CNTGO@MFGO	0.7941	0.0622	1.1639	0.1147
$C_{60}(OH)_{26-32}$@MFGO	0.8231	0.2804	1.3796	0.6969

S5 Calculation of the dynamic water vapor permeance

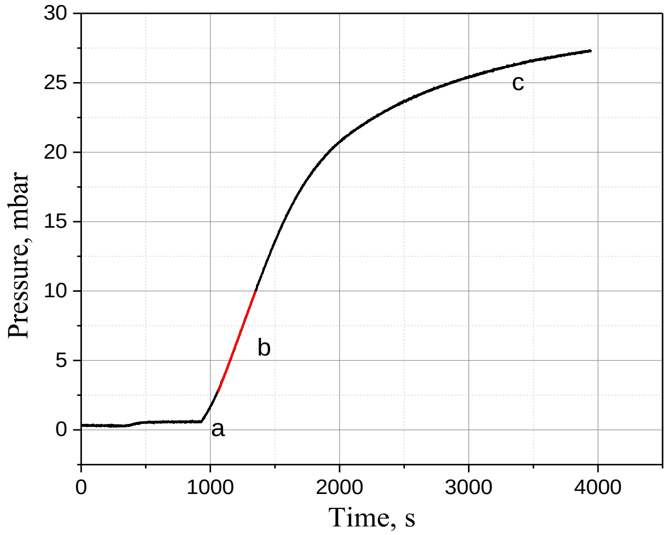

Figure S5. The pressure-time dependence (curve) registered in the membrane permeate site in the dynamic wet N_2 (nearly 100% RH) transport across composite membrane. The part of the curve under the latter “a” denotes the period of equilibrium establishment of the wet N_2 flux through a membrane when the permeate chamber is evacuated by a pump; the part of the curve under the letter “b” (marked with red) represents the gradual pressure increase in the permeate chamber of the measurement cell; the part of the curve under the letter “c” shows the pressure level achieving saturated H_2O vapors pressure.

The water vapor flux in a dynamic mode is calculated using the tangent of the part of the curve under the letter “b” (marked with red). The water vapor permeance was calculated according to the formula:

$$P_{H2O} = \frac{F_{H2O}}{S \cdot \Delta p_{H2O}}$$

where F_{H2O} is the water vapor flux, m^3/h; S – membrane area, m^2; Δp_{H2O} – the difference in partial pressure of water vapours at the start and end of the linear part of the pressure-time curve.

References

(1) *NIST X-ray Photoelectron Spectroscopy Database, NIST Standard Reference Database Number 20, National Institute of Standards and Technology, Gaithersburg MD, 20899.*
